# Hypertension and Stroke Cardiovascular Control Evaluation by Analyzing Blood Pressure, Cerebral Blood Flow, Blood Vessel Resistance and Baroreflex

**DOI:** 10.3389/fbioe.2021.731882

**Published:** 2021-12-10

**Authors:** Shoou-Jeng Yeh, Chi-Wen Lung, Yih-Kuen Jan, Fang-Chuan Kuo, Ben-Yi Liau

**Affiliations:** ^1^ Section of Neurology and Neurophysiology, Cheng-Ching General Hospital, Taichung, Taiwan; ^2^ Department of Creative Product Design, Asia University, Taichung, Taiwan; ^3^ Rehabilitation Engineering Lab, Kinesiology and Community Health, Computational Science and Engineering, University of Illinois at Urbana-Champaign, Champaign, IL, United States; ^4^ Department of Physical Therapy, Hungkuang University, Taichung, Taiwan; ^5^ Department of Biomedical Engineering, Hungkuang University, Taichung, Taiwan

**Keywords:** cardiovascular control, hypertension, stroke, blood pressure, cerebral blood flow, blood vessel resistance, baroreflex

## Abstract

Cardiovascular diseases have been the leading causes of mortality in Taiwan and the world at large for decades. The composition of cardiovascular and cerebrovascular systems is quite complicated. Therefore, it is difficult to detect or trace the related signs of cardiovascular and cerebrovascular diseases. The characteristics and changes in cardiopulmonary system disease can be used to track cardiovascular and cerebrovascular disease prevention and diagnosis. This can effectively reduce the occurrence of cardiovascular and cerebrovascular diseases. This study analyzes the variability in blood pressure, cerebral blood flow velocity and the interaction characteristics using linear and nonlinear approaches in stroke, hypertension and healthy groups to identify the differences in cardiovascular control in these groups. The results showed that the blood pressure and cerebral blood flow of stroke patients and hypertensive patients were significantly higher than those of healthy people (statistical differences (*p* < 0.05). The cerebrovascular resistance (CVR) shows that the CVR of hypertensive patients is higher than that of healthy people and stroke patients (*p* < 0.1), indicating that the cerebral vascular resistance of hypertensive patients is slightly higher. From the patient’s blood flow and vascular characteristics, it can be observed that the cardiovascular system is different from those in healthy people. Baroreflex sensitivity (BRS) decreased in stroke patients (*p* < 0.05). Chaotic analysis revealed that the blood pressure disturbance in hypertensive patients has a higher chaotic behavior change and the difference in initial state sensitivity. Cross-correlation (CCF) analysis shows that as the course of healthy→hypertension→stroke progresses, the maximum CCF value decreases significantly (*p* < 0.05). That means that blood pressure and cerebral blood flow are gradually not well controlled by the self-regulation mechanism. In conclusion, cardiovascular control performance in hypertensive and stroke patients displays greater variation. This can be observed by the bio-signal analysis. This analysis could identify a measure for detecting and preventing the risk for hypertension and stroke in clinical practice. This is a pilot study to analyze cardiovascular control variation in healthy, hypertensive and stroke groups.

## 1 Introduction

Cardiovascular diseases (CVD) have become a leading health care burden in many areas of the world. It was reported that high blood pressure is the main risk factor to induce CVD. Most deaths are caused by ischemic heart disease and ischemic stroke (Roth et al., 2020). Therefore, systolic blood pressure variability could be assessed as a stroke and CVD risk predictor in the hypertensive population (Pringle et al., 2003). Because CVD is common in most areas of the world, WHO established a CVD risk prediction chart to reduce the medical burden (WHO CVD risk chart working group, 2019). Moreover, blood pressure is highly related to CVD (Fuchs and Ethlton, 2020). Hypertension is high blood pressure in the blood vessels. In the brain, high blood pressure induces hypertrophy and remodels smooth muscle cells in the cerebral arteries (Yu et al., 2011). The changes in blood vessel wall composition leads to greater cerebral artery stiffness. Aortic stiffness could be an independent predictor of hypertensive-stroke patient (Baumbach et al., 1988; Laurent et al., 2003, Laurent et al., 2005; Benjo et al., 2007). The change in blood pressure is the earliest sign of abnormal cardiopulmonary circulation. The change in blood pressure causes considerable variation in the physiological feedback mechanism. The relationship between blood pressure and cerebral blood flow in the brain is cerebral autoregulation (CA). Cerebral autoregulation maintains cerebral blood flow to protect the brain by reducing the effect of blood pressure variation. Previous studies reported that impaired CA may be associated with higher stroke risk (Shekhar et al., 2017; Castro et al., 2018). Therefore, CA assessment is also important to predict and reduce CVD risk. CA measurement and evaluation may obtain a predictive value for the development of delayed cerebral ischemia and radiographic vasospasm (Tiecks et al., 1995; Aoi wt al., 2012; Oeinck et al., 2013; Ma et al., 2016; Crippa et al., 2018). Although some non-invasive approaches (ex. Cross-correlation function, chaotic analysis etc.) and devices (ex. Near-infrared spectroscopy, transcranial Doppler etc.) have been developed to assess CA, measuring CA is difficult and standard measurement is not currently available (Tiecks et al., 1995; Liau et al., 2010; Rivera-Lara et al., 2017; Xiong et al., 2017). On the other hand, few studies revealed the differences in physiological signals and properties between hypertension and stroke. The changes in the cardiovascular disease physiological parameter development process (healthy→hypertension→stroke) are not clear. The objective of this study was to apply linear and nonlinear physiological signal analysis methods to assess multiple blood pressure signal correlation effects on cerebral blood flow signals, blood vessel properties, baroreflex and CA in healthy people, hypertension and stroke patients. To the best of our knowledge, this is the first study to investigate bio-signal performance and differences. The findings from multiple views could be used to better understand the effects of various cardiovascular diseases on bio-signal variation and tissue properties as assessed using multi-correlation approaches.

## 2 Materials and Methods

### 2.1 Subjects and Measurement

In this study, 3 groups were enrolled: 1) 11 healthy subjects (57.4 ± 8.4 years) that have no history of related cardiovascular diseases. 2) 11 hypertensive patients (50.8 ± 10.3 years) from the Neurology Section of Cheng-Ching General Hospital, Taiwan. Hypertension was according to WHO, 2003 that clinic blood pressure ≥140/90 mmHg (WHO, 2003). 3) 10 hypertensive stroke outpatients (56 ± 10.16 years) from the Neurology Section of Cheng-Ching General Hospital were enrolled in this study. These stroke patients have to qualify blood pressure level defined as a clinic blood pressure ≥140/90 mmHg (WHO, 2003). National Institutes of Health Stroke Scale, NIHSS <15. Stroke more than 7 days. The subjects in 3 groups were age-matched and none of the subjects were receiving any medication during the study period. Informed consent was received from all subjects prior to entry into the study. This study was approved by the Research Ethics Committee of Cheng-Ching General Hospital, Taiwan. Continuous arterial blood pressure signals were acquired via using the Finapres (Model 2,300, Ohemda, Englewood, CO, United States). Cerebral blood flow velocity signals were obtained through TCD (transcranial Doppler ultrasound, EME TC 2020, Nicolet instrument, Warwick, United Kingdom) in conjunction with a 5-MHz transducer fixed over the temporal bones by an elastic headband. Subjects lied down on a tilt-table that enabled a motor-driven change from a supine to an upright position at 75° within 4 s. Data acquisition was started after a 10-min relaxation period in the supine position. After that, continuous arterial blood pressure and cerebral blood flow velocity signals were acquired during both supine and 75° head-up tilt positions and then returned to supine and rest for 5 minutes. The experimental devices included a general-purpose data acquisition board with a computer and LabVIEW program for acquiring signal processing. This equipment was developed in our previous study (Chiu et al., 2007; Liau et al., 2010).

### 2.2 BP and CBFV Signals

**FIGURE 1:**
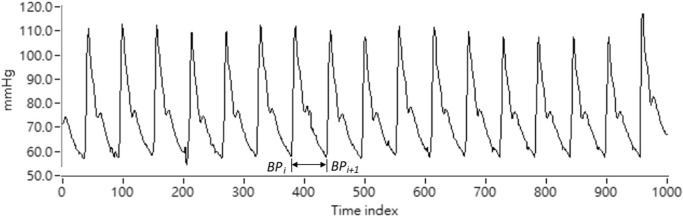
Representation of the ABP signal.

**FIGURE 2:**
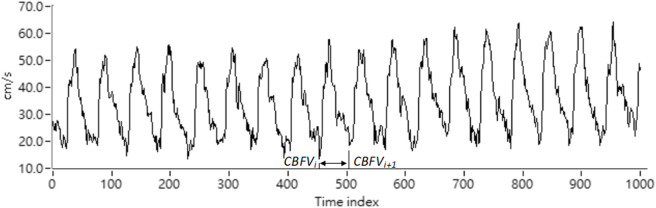
Representation of the CBFV signal.

Mean value estimation is based on every waveform peak and valley location. The mean arterial blood pressure (MABP) value was calculated using each pulse as follows:

$${\mathrm{MABP}}_{i}=\frac{1}{P_{i+1}-P_{i}}\sum_{k=P}^{P_{i+1}}\mathrm{BP}\left(k\right)$$ (1)

where *BP*(*‧*)in Eq. 1 is the arterial blood pressure pulse signal acquired by Finapres continuously. *P*_*i*_ is the wave-through time index in the *i*th BP pulse beat. Therefore, *MABP*_*i*_ is the mean BP value calculated by the *i*th pulse beat. Representation of the BP signal is shown as Figure 1.On the other hand, mean cerebral blood flow velocity (MCBFV) could be obtained using Eq. 2 as follows.

$${\mathrm{MCBFV}}_{i}=\frac{1}{V_{i+1}-V_{i}}\sum_{k=V}^{V_{i+1}}\mathrm{CBFV}\left(k\right)$$ (2)

where *CBFV(‧)* is the CBFV pulse signal continuously acquired by the TCD. *Vi* is the time index of the wave-through in the CBFV signal corresponding to the *i*th pulse beat. *MCBFVi* is the mean CBFV value for the *i*th pulse beat. The CBFV signal representation is shown as Figure 2.

### 2.3 Methodology

#### 2.3.1 Resistance Index

Resistance index is a measure of peripheral blood flow resistance. Low vascular resistance has a higher diastolic blood flow velocity characteristic, and will have a lower RI. A high vascular resistance has a lower diastolic blood flow velocity characteristic, and will produce a higher RI. RI can be calculated using the following formula, SCBFV is the systolic blood flow velocity, and DCBFV is the diastolic blood flow velocity. RI can be obtained by Eq. 3 as below (Hilz et al., 2004).

$$\text{RI}=\frac{\mathrm{SCBFV}-\mathrm{DCBFV}}{\mathrm{SCBFV}}$$ (3)

#### 2.3.2 Pulsatility Index

The pulsation index is a measurement that describes the type of signal waveform. Low intracranial vascular resistance will reduce PI, and rising intracranial pulsations have been found to be related to rising intracranial pressure. The normal PI range is between 0.5 and 1.4, less than 0.5 may be an ischemic flow pattern under vascular dilation, and greater than 1.5 may be a decrease in vascular compliance or an increase in intracranial pressure. PI can be calculated using the following formula, SCBFV is the systolic blood flow velocity, DCBFV is the diastolic blood flow velocity, and MCBFV is the average blood flow velocity. *PI* can be obtained by Eq. 4 (Hilz et al., 2004)

$$\text{PI}=\frac{\text{SCBFV}-\text{DCBFV}}{\text{MCBFV}}$$ (4)

#### 2.3.3 Cerebrovascular Resistance

The cerebrovascular resistance can be expressed as the ratio of the average pressure to the average cerebral blood flow rate. The unit is mmHg/(cm/s). CVR can be calculated using the following formula, where MABP is the average blood pressure and MCBFV is the average cerebral blood flow. *CVR* can be obtained using Eq. 5 (Hilz et al., 2004)

$$\mathrm{CVR}=\frac{\mathrm{MABP}}{\mathrm{MCBFV}}$$ (5)

#### 2.3.4 Cross-Correlation Analysis

Cross-correlation analysis mainly observes the correlation between the two signals in the time domain. Taking the average cerebral blood flow signal and the average blood pressure signal as an example, the correlation and phase relationship between the average cerebral blood flow signal and the average blood pressure signal can be determined by the brain. The phase relationship between blood flow and blood pressure is used to explore the blood flow regulation mechanism using the cardiovascular system. Let the cross-correlation function be expressed as CCF(k), W is the length of the window, k is the number of peak-to-peak displacement points, N is the total length of the signal, and the MABP time series normalized with the average value is expressed as x(n), MCBFV time series is expressed as y(n), the time series of x(n) after band-pass filtering is expressed as, and the time series of y(n) after band-pass filtering is expressed as, then cross-correlation The analyzed formula is as follows (Chiu et al., 2001; Chiu et al., 2005; Liau et al., 2010; Fan et al., 2019):

$${\mathrm{CCF}}_{i}\left(k\right)=\frac{R_{\hat{x}\hat{y}}^{i}\left(k\right)}{{\left[R_{\hat{x}\hat{x}}^{i}\left(0\right)R_{\hat{y}\hat{y}}^{i}\left(0\right)\right]}^{\frac{1}{2}}},\;k=0,\;\pm 1,\;\pm 2,\ldots ,\;i=1\;\text{to}\;N-W+1$$ (6)

where

$$\begin{array}{l} R_{\hat{x}\hat{y}}^{i}\left(k\right)=\{\begin{array}{c} \frac{1}{W}\sum_{j=i}^{i+W}\hat{x}\left(j\right)\hat{y}\left(j+k\right),\;k=0,1,2,\ldots \\ \frac{1}{W}\sum_{j=i}^{i+W}\hat{x}\left(j-k\right)\hat{y}\left(j\right),\;k=0,-1,-2,\ldots \end{array}\\ R_{\hat{x}\hat{x}}^{i}\left(0\right)=\frac{1}{W}\sum_{j=i}^{i+W}{\left[\hat{x}\left(j\right)\right]}^{2},\text{and}\;R_{\hat{y}\hat{y}}^{i}\left(0\right)=\frac{1}{W}\sum_{j=i}^{i+W}{\left[\hat{y}\left(j\right)\right]}^{2} \end{array}$$

#### 2.3.5 Baroreflex Sensitivity

The index is used to evaluate the pressure-sensitive reflex. In order to observe the changes in heart rate and blood pressure at the same time, the baroreflex sensitivity index observes the relationship between the heart rate signal and the blood pressure signal. Assuming that the systolic blood pressure sequence SBP is S(n), and the heartbeat interval RR interval is R(n), the baroreflex sensitivity (BRS) T(n) can be expressed as: (Karemaker and Wesseling, 2008):

$$T\left(n\right)=\sum_{k=1}^{n}\frac{R\left(k\right)}{S\left(k\right)}$$ (7)

#### 2.3.6 Correlation Dimension

The correlation dimension (CD) can quantify the properties of the attractors from the time series and determine the singularity of the attractors, which is estimated by the correlation function by calculating the pairs of points where the attractors fall on the radius of the “sphere”. The correlation function Cd(R) is defined as (Grassberger and Procaccia, 1983; Liau et al., 2008; Gao et al., 2011; Bolea et al., 2014):

$$C_{d}\left(R\right)=\underset{N\to \infty }{\lim}\left[\frac{1}{N^{2}}\sum_{i,j=1,i\neq j}H_{E}\left(R-\left|X_{i}-X_{j}\right|\right)\right]$$ (8)

*N*:number of points
*H*_*E*_:Heaviside step function
*H*_*E*_ = 1, if *R*-| *X*_*i*_- *X*_*j*_ | ≧ 0
*H*_*E*_ = 0, otherwise
(*X*_*i*_, *X*_*j*_)The Euclidean distance of an attractor in 1 dm dimension is:

$$\left|X_{i}-X_{j}\right|={\left[{\left(x_{i,1}-x_{j,1}\right)}^{2}+{\left(x_{i,2}-x_{j,2}\right)}^{2}+\cdots {\left(x_{i,dm}-x_{j,dm}\right)}^{2}\right]}^{1/2}$$

The relationship between the correlation integral and R is: Cd(R)∼R^dc^, the logarithm of both sides can be obtained:

$$\log C_{d}\left(R\right)=d_{c}\log R+\text{constant}$$

#### 2.3.7 Lyapunov Exponent

The Lyapunov exponent (LE) is used to quantitatively analyze the chaotic behavior by measuring the sensitivity of the attractor initial state. Its physical meaning is the exponential rate of the divergence of adjacent trajectories in the attractor. It is used to measure the degree of attraction or separation between two adjacent trajectories in phase space. For example: the initial distance between two points of adjacent trajectories is *d*_*L*_ (0), and then the distance becomes *d*_*L*_(t) due to the trajectory divergence at time t, so $d_{L}\left(t\right)=d_{L}\left(0\right)e^{\lambda t}$ . A positive LE value indicates that the system dynamic behavior is chaotic, and a negative value or zero indicates regular behavior. For an N-dimensional attractor, there will be N LE values. The algorithm for calculating LE is to obtain the maximum positive LE (that is, *λ*_1_) in the time series. This method calculates the divergence rate of the reference trajectory and adjacent trajectories in the attractor. The maximum positive LE of the time series can be expressed as (Eckhardt and Yao, 1993; Rozenbaum et al., 2017):

$$\lambda_{1}=\underset{N\to \infty }{\lim}\frac{1}{t_{m}-t_{0}}\sum_{i=1}^{m}\ln\frac{d_{L}^{'}\left(t_{i}\right)}{d_{L}\left(t_{i-1}\right)}$$ (9)

*t*_*0*_:initial time*t*_*m*_:the *m*th time.

#### 2.3.8 Kolmogorov Entropy (K2)

Kolmogorov entropy is a quantitative method for measuring the degree of chaotic behavior. It can also be used to distinguish the characteristics of dynamic systems: chaotic or non-chaotic. “Entropy” is adopted from the concept of thermodynamics as a method for expressing information characteristics, that is, the prediction of an uncertain system. The Kolmogorov entropy concept can be expressed as:

$$S\left(t_{2}\right)=S\left(t_{1}\right)+K\left(t_{2}-t_{1}\right)$$ (10)

*K* stands for Kolmogorov entropy. The unit is bit/s. *S*(*t*_*1*_ *,t*_*2*_) is the amount of change in the time development information predicted by the initial information *S*(*t*_*1*_) after one (t_2_—t_1_) time. Assume that the initial entropy information is *S*(*t*_*1*_), after a time interval *t*_*2*_ *—t*_*1*_, the information change to become *S*(*t*_*2*_). The change difference information is *K*(*t*_*2*_ *—t*_*1*_). Thus, it can be derived: *S*(*t*_*2*_) *= S*(*t*_*1*_) *+ K*(*t*_*2*_ *—t*_*1*_)*.* When *S*(*t*_*1*_) *> S*(*t*_*2*_), so *K*(*t*_*2*_ *—t*_*1*_) *= S*(*t*_*1*_)*—S*(*t*_*2*_). Assume *S*(*t*_*1*_) = ln*C*_*d*_(*R*), *S*(*t*_*2*_) = ln*C*_*d+1*_(*R*). *C*_*d*_(*R*) is the signal correlation function. The equation for *K2* is derived as below (Grassberger 1983; Zhang 2017):

$$K2=\ln\frac{{\text{C}}_{d}\left(R\right)}{C_{d+1}\left(R\right)}$$ (11)

*t-test* was used to determine the significance of difference between healthy group and patient groups. The significance level was set to 0.05. All statistical tests were performed using SPSS 26 (IBM, Somers, NY, United States).

## 3 Results

### 3.1 Blood Pressure and Cerebral Blood Flow

**FIGURE 3:**
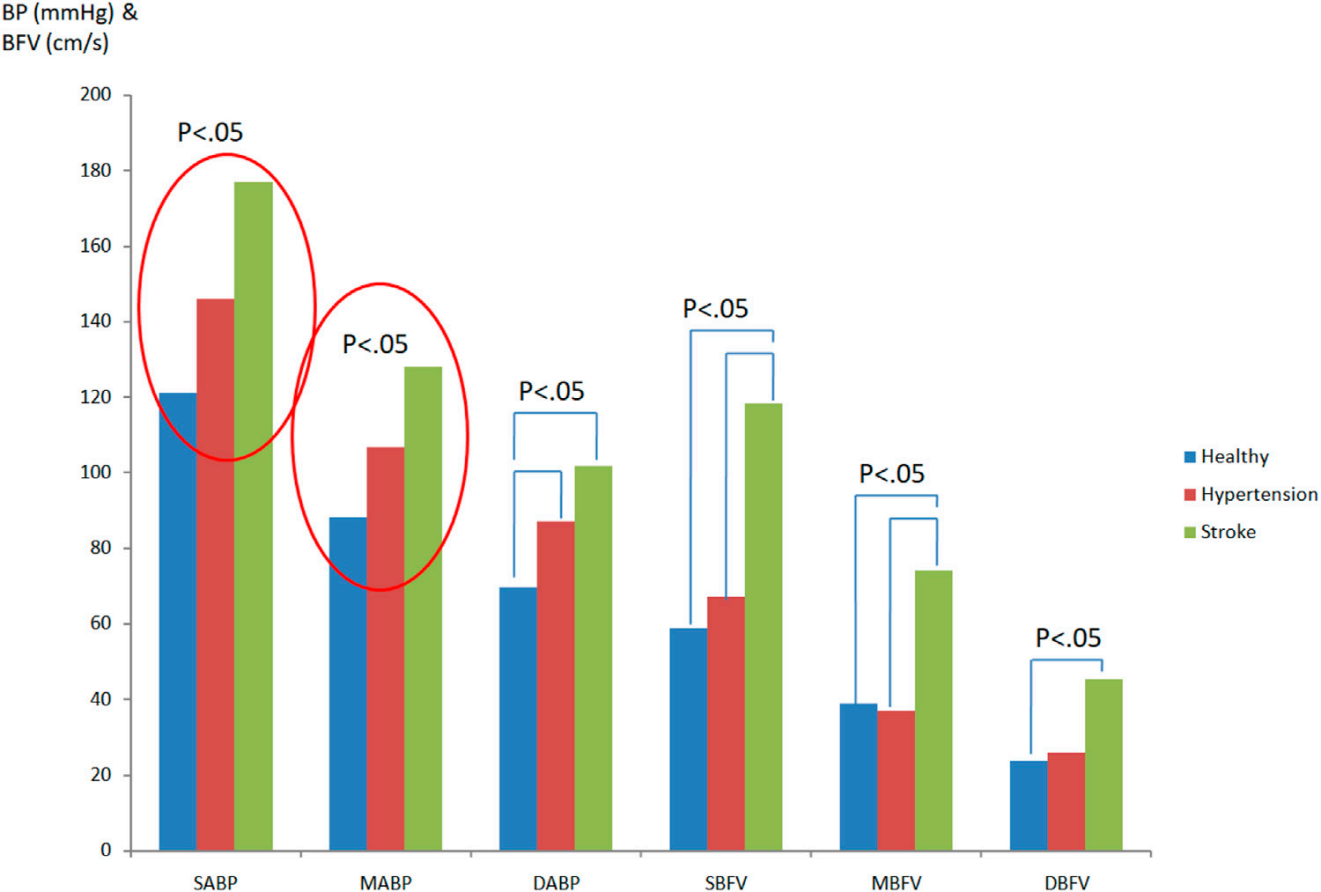
Comparison of differences in blood pressure and cerebral blood flow while in supine position among groups.

Figure 3 showed the comparison of differences in blood pressure and cerebral blood flow while in supine position among groups. From the systolic arterial blood pressure (SABP) and mean arterial blood pressure (MABP), it can be observed that stroke patients (SABP:177.0 ± 21.25 mmHg; MABP: 127.95 ± 18.66 mmHg)>hypertension patients (SABP:146.07 ± 26.04 mmHg; MABP: 106.55 ± 19.72 mmHg)>healthy people (SABP:120.94 ± 7.74 mmHg; MABP: 88.11 ± 7.76 mmHg). Both of these differences were significant (*p* < 0.05). Diastolic arterial blood pressure (DABP) is statistically different in stroke patients (DABP: 101.69 ± 18.02 mmHg) as opposed to hypertensive patients (DABP: 87.01 ± 18.16 mmHg) (*p* < 0.05). It can be seen that cardiovascular diseases (hypertension, stroke) do have obvious changing factors and trends to increase blood pressure. The systolic cerebral blood flow velocity (SCBFV) and mean cerebral blood flow velocity (MCBFV) showed that the value of stroke patients (SCBFV: 118.37 ± 60.42 cm/s; MCBFV: 73.99 ± 39.20 cm/s) was significantly higher than that of hypertension patients (SCBFV: 67.19 ± 21.84 cm/s; MCBFV: 36.84 ± 16.55 cm/s) and healthy people (SCBFV: 58.73 ± 9.62 cm/s; MCBFV: 38.82 ± 7.84 cm/s), and there was a statistical difference (*p* < 0.05). Diastolic cerebral blood flow velocity (DCBFV) is statistically different between stroke patients (DCBFV: 45.12 ± 24.78 cm/s) and healthy people (DCBFV: 23.69 ± 6.90 cm/s) (*p* < 0.05), and there is no significant difference in diastolic cerebral blood flow between hypertensive patients and stroke patients. Figure 3 indicated the significant differences in blood pressure and cerebral blood flow while in supine position among healthy people, hypertensive patients, and stroke patients.

**FIGURE 4:**
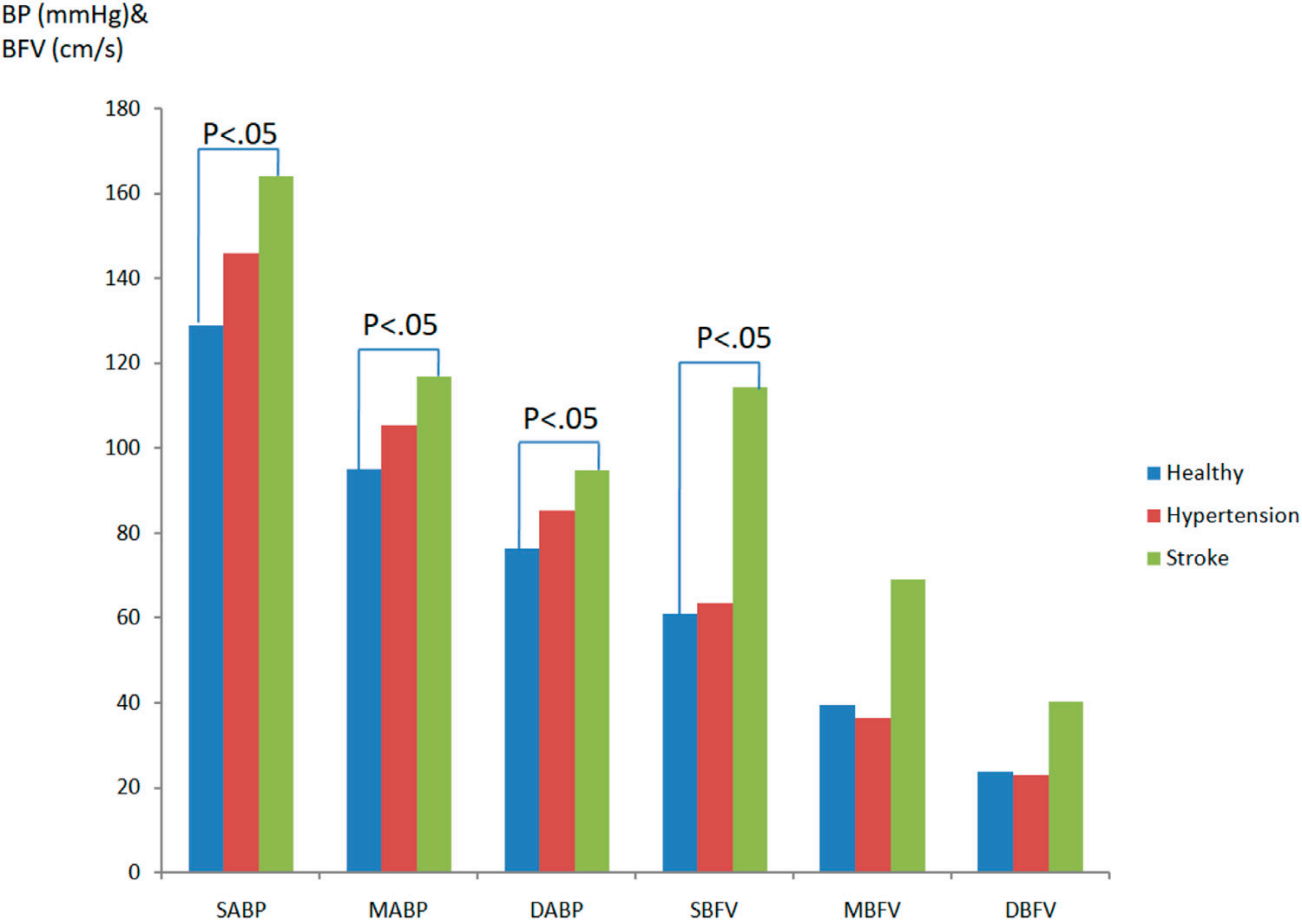
Blood pressure and cerebral blood flow differences comparison in healthy people, hypertensivepatients, and stroke patients in head-up tilt position.

Figure 4 revealed the blood pressure and cerebral blood flow differences comparison in healthy people, hypertensive patients, and stroke patients in head-up tilt position. After interference induced by the tilting table, it can be seen that the SABP, MABP, and DABP values of stroke patients are significantly (*p* < 0.05) higher than those in healthy people (SABP_stroke_:163.97 ± 19.55 mmHg; MABP_stroke_:116.90 ± 12.78 mmHg; DABP_stroke_:94.71 ± 12.38 mmHg; SABP_healthy_:128.83 ± 18.18 mmHg; MABP_healthy_:95.0 ± 11.79 mmHg; DABP_healthy_:76.30 ± 11.49 mmHg). On the other hand, the BP values of hypertensive patients between healthy and stroke patients were without significant difference and this might indicate an increasing BP trend toward abnormal. The cerebral blood flow velocity of stroke patients is higher than that of healthy people. The systolic cerebral blood flow velocity values revealed a significant statistical difference (SCBFV_stroke_:114.18 ± 60.65 cm/s; SCBFV_healthy_:60.87 ± 14.05 cm/s, *p* < 0.05).

### 3.2 Resistance Index, Pulsation Index) and Cerebrovascular Resistance

**FIGURE 5:**
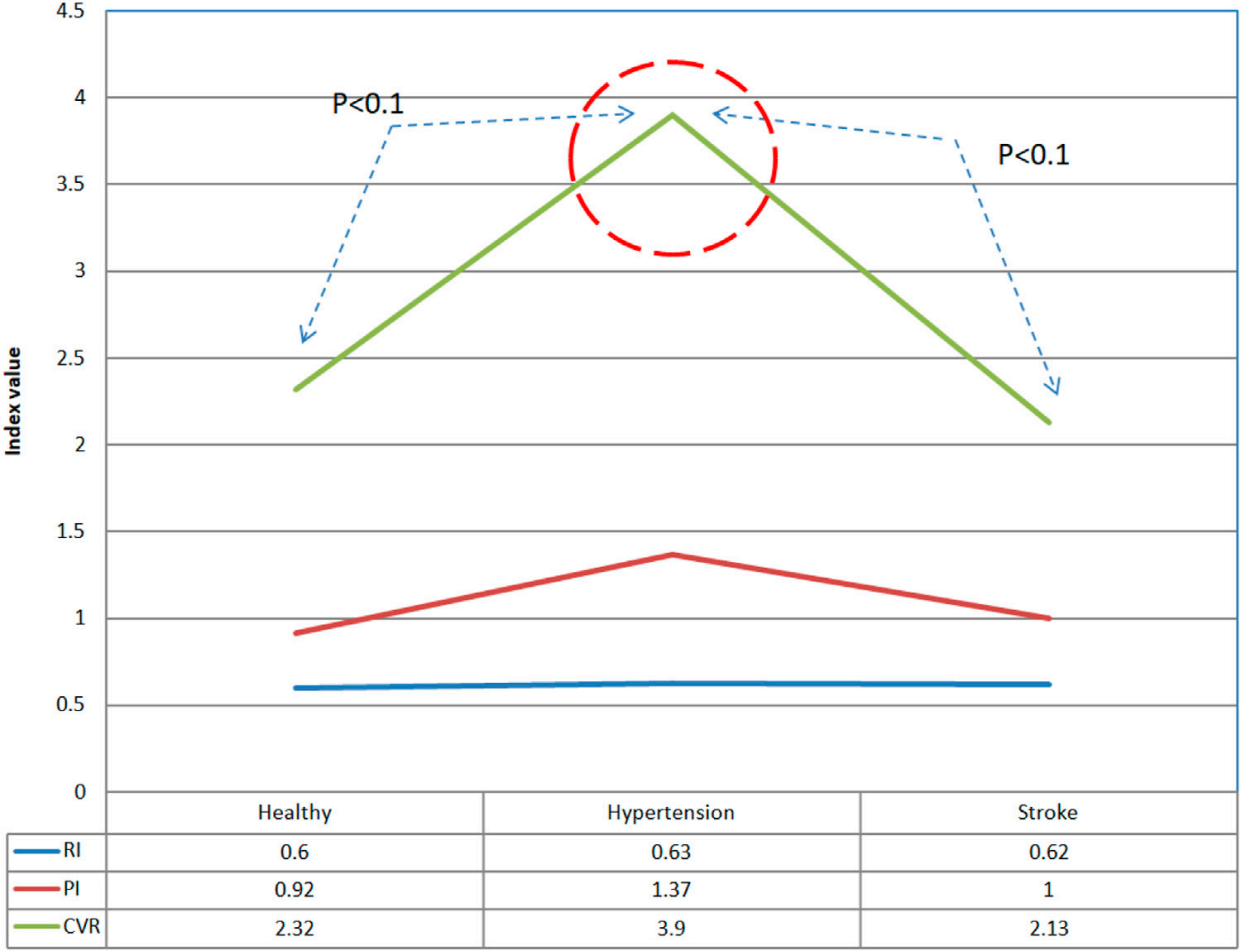
Comparison of differences in vascular resistance indexes between healthy people, hypertension patients, and stroke patients in supine position.

**FIGURE 6:**
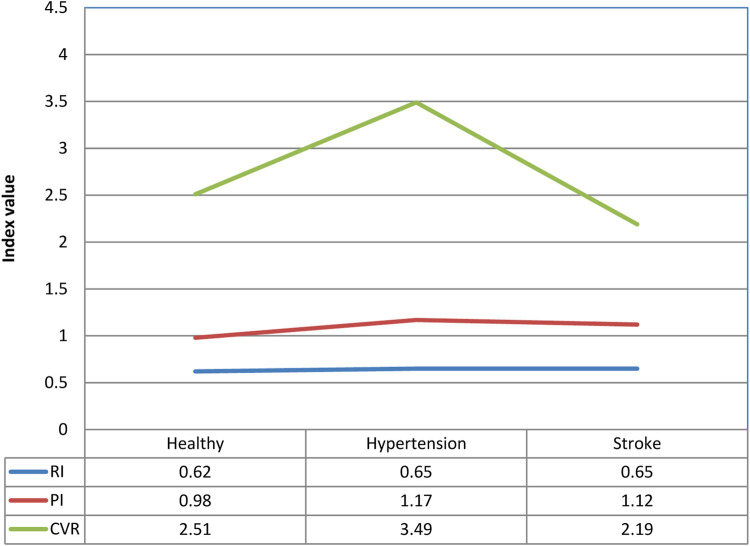
Comparison of differences in vascular resistance indexes of healthy people, hypertensive patients, and stroke patients in tilting position.

Figures 5, 6 show the resistance index (RI), pulsation index (PI), and cerebrovascular resistance (CVR) analysis results of the three groups in both supine and tilting positions, respectively. There is no obvious difference in movement and progress in RI and PI during position change. The CVR values indicated a slight statistical difference between healthy people and hypertensive patients (CVR_healthy_: 2.32 ± 0.32; CVR_hypertension_: 3.9 ± 2.78), hypertensive patients vs. stroke patients (CVR_hypertension_: 3.9 ± 2.78; CVR_hypertension_: 2.13 ± 1.03) while in supine position (*p* < 0.1). There was no significant difference in PI in the subjects tested this time. Only the PI values of hypertensive patients and stroke patients tended to be slightly higher than normal. The cerebrovascular resistance index CVR shows that the CVR of hypertensive patients is higher than that of normal people and stroke patients (*p* < 0.1), which shows that the cerebral vascular resistance of hypertensive patients is higher, but it is speculated that once a stroke develops, the cerebral vascular lesions Instead, it reduces the vascular resistance in the brain.

### 3.3 Analysis of Baroreflex

**FIGURE 7:**
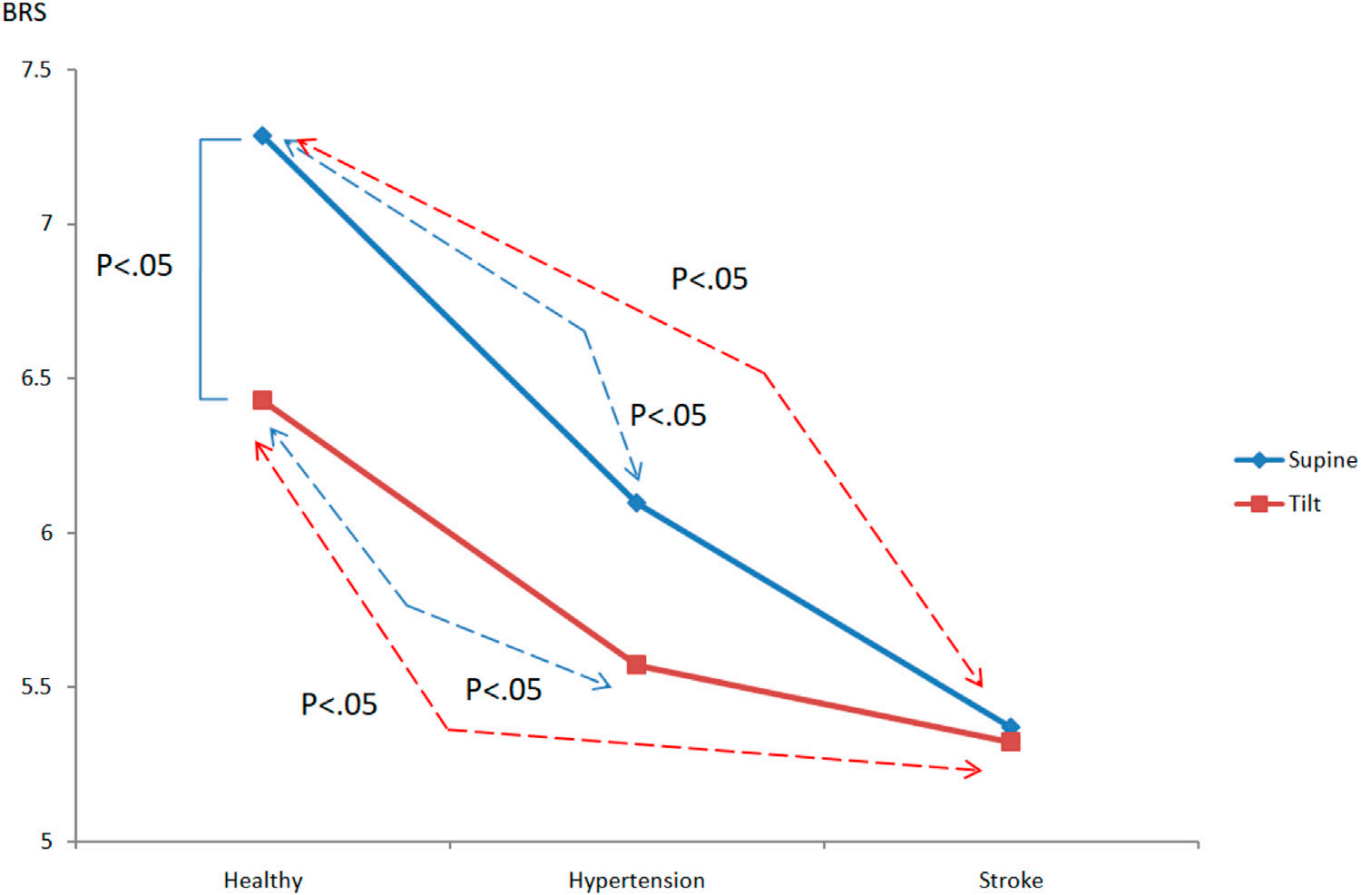
Comparison of the differences in pressure reflex between healthy people, hypertensive patients, and stroke patients.

The pressure-sensitive reflex degree—baroreflex sensitivity (BRS) (also known as the pressure-sensitive reflex gain) is used as a measure of the autonomous control of the cardiovascular system. Usually BRS is a measure of the response of autonomic effectors to a given change in arterial pressure. When the position of a person changes from supine to a head-up position, blood pressure drops. This decreasing blood pressure can be compensated by the baroreflex through the conduction of the vagus nerve reflex. The sensory signal caused by the pressure receptor can inhibit the activity of the parasympathetic nerve and promote the activity of the sympathetic nerve, which increases the heart rate and vasoconstriction, helping to maintain proper blood pressure when standing. Figure 7 shows the baroreflex sensitivity analysis results of healthy people, hypertensive patients, and stroke patients. It shows that as the course of healthy→hypertension→stroke progresses, BRS_healthy_ > BRS_hypertension_ > BRS_stroke_, and BRS decreases accordingly. Healthy people vs. hypertension (supine: BRS_healthy_: 7.29 ± 0.88, BRS_hypertension_: 6.1 ± 1.49; tilting: BRS_healthy_: 6.43 ± 0.98, BRS_hypertension_: 5.57 ± 0.86), healthy people vs. stroke (supine: BRS_healthy_: 7.29 ± 0.88, BRS_stroke_: 5.37 ± 1.33; tilting: BRS_healthy_: 6.43 ± 0.98, BRS_stroken_: 5.32 ± 1.22), there are significant differences in both supine and tilting positions (*p* < 0.05).

### 3.4 Nonlinear Blood Pressure and Cerebral Blood Flow Analysis

**FIGURE 8:**
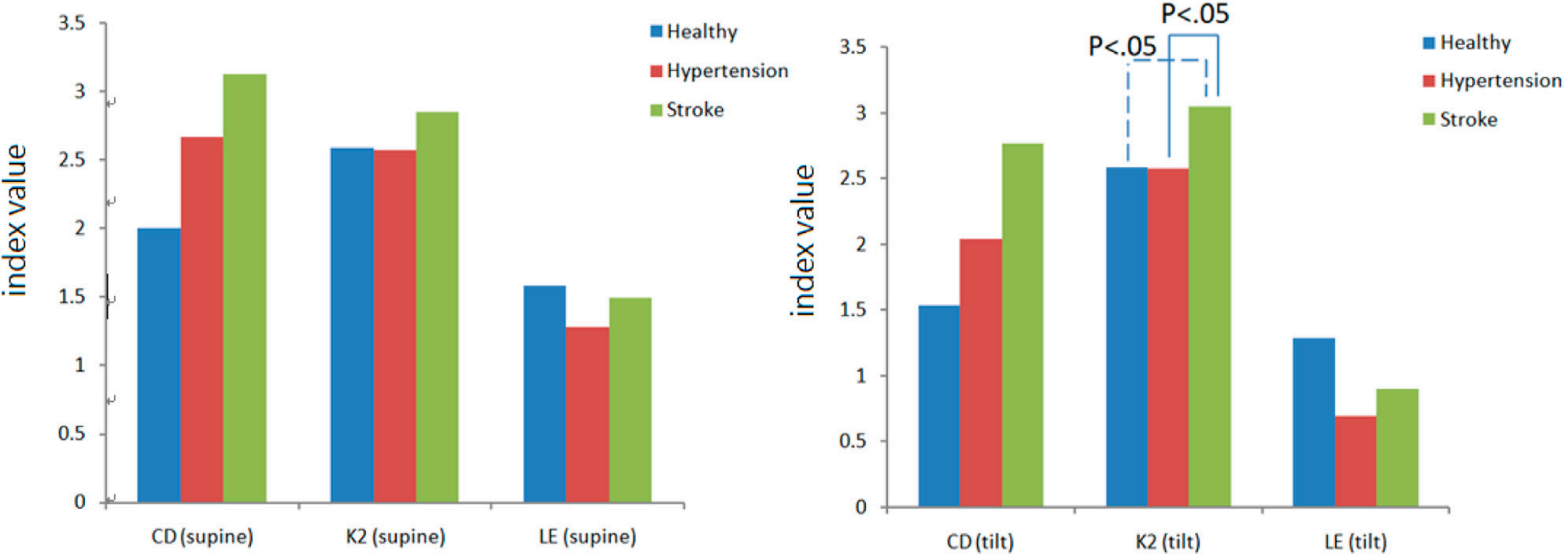
Differences in blood pressure chaotic analysis of healthy people, hypertensive patients, and stroke patients.

**FIGURE 9:**
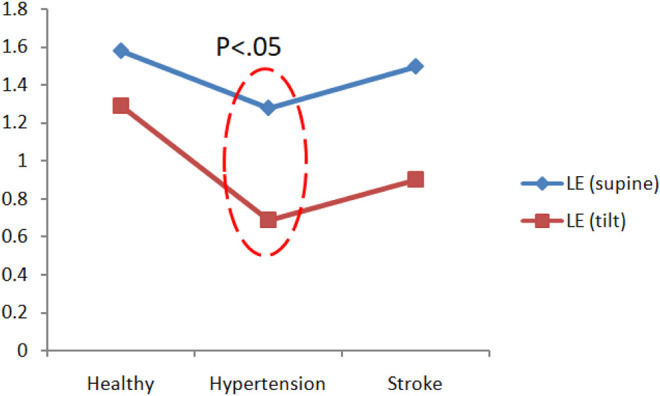
Comparison of blood pressure Lyapunov exponent (LE) analysis of healthy people, hypertension patients, and stroke patients.

There are three main parameters in chaotic analysis: 1. Correlation dimension (CD) represents the complexity of a system, and the higher the CD value, the higher the system complexity. 2. Lyapunov exponent (LE) represents the sensitivity of the initial system state. A positive LE value indicates that the system dynamic behavior is chaotic, and a negative value or zero indicates regular behavior. 3. Kolmogorov entropy (K2) is a quantitative method for the degree of chaotic behavior. It is used to predict the information loss rate of future behavior, that is, the degree of unpredictability. For a regular system, K2 is zero. For a completely random system, the K2 value is infinite. For a chaotic system, the K2 value is finite and positive. Figure 8 is a comparison chart of the differences in blood pressure chaos analysis between healthy people, hypertensive patients, and stroke patients. It can be observed that the change trend between supine and head-up positions is close. After head-up tilting, the K2 value of stroke patients is greater than that of hypertension patients and healthy people (K2_stroke_:3.04 ± 0.28, K2_hypertension_:2.58 ± 0.39, K2_healthy_:2.59 ± 1.11), and there are statistical differences (*p* < 0.05) as shown in Figure 8. On the other hand, when the LE value changes posture in hypertensive patients, there is a statistically significant difference (Supine-LE_hypertension_:1.28 ± 0.67,Tilt-LE_hypertension_:0.69 ± 0.43*p* < 0.05), such as shown in Figure 9.

**FIGURE 10:**
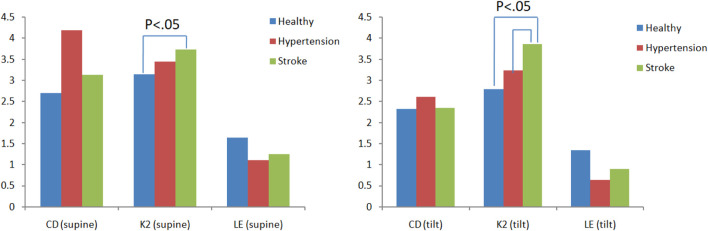
Comparison of differences in cerebral blood flow chaos analysis among healthy people, hypertensive patients, and stroke patients.

Figure 10 is a comparison chart of the chaotic analysis of cerebral blood flow in healthy people, hypertensive patients, and stroke patients. It can be observed that there is a significant difference (*p* < 0.05) in the K2 index between supine and tilting positions. While in supine position, the K2 value of stroke was higher than that of healthy people, with a statistical difference (K2_stroke_: 3.74 ± 0.42, K2_healthy_: 3.14 ± 0.59, *p* < 0.05). After head-up tilting, the K2 values ​​of stroke patients were higher than those in hypertension patients and healthy people, and there was a statistical difference (K2_stroke_:3.87 ± 1.03; K2_hypertension_:3.24 ± 0.3; K2_healthy_:2.80 ± 0.46, *p* < 0.05) as shown in Figure 10. When the LE value during changes posture in hypertensive patients, there is a statistically significant difference (supine LE_hypertension_:1.11 ± 0.43; tilting LE_hypertension_:0.64 ± 0.25, *p* < 0.05).

### 3.5 Cross-Correlation Function Analysis

#### 3.5.1 Blood Pressure and Cerebral Blood Flow

**FIGURE 11:**
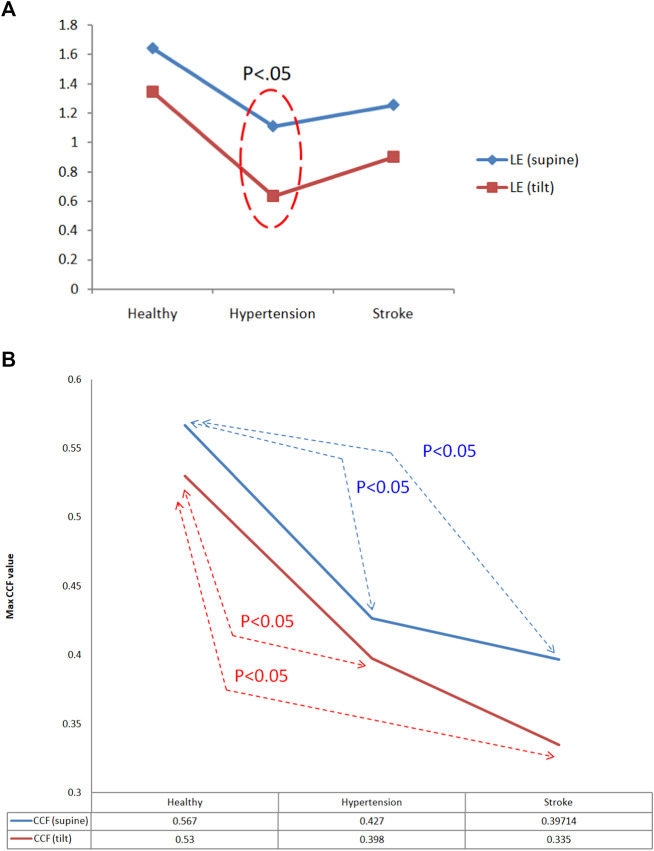
**(A)** Comparison of Lyapunov exponent (LE) analysis of cerebral blood flow in healthy people, hypertensive patients, and stroke patients. **(B)**Comparison of maximum CCF differences between blood pressure and cerebral blood flow in healthy people, hypertensive patients, and stroke patients.

**FIGURE 12:**
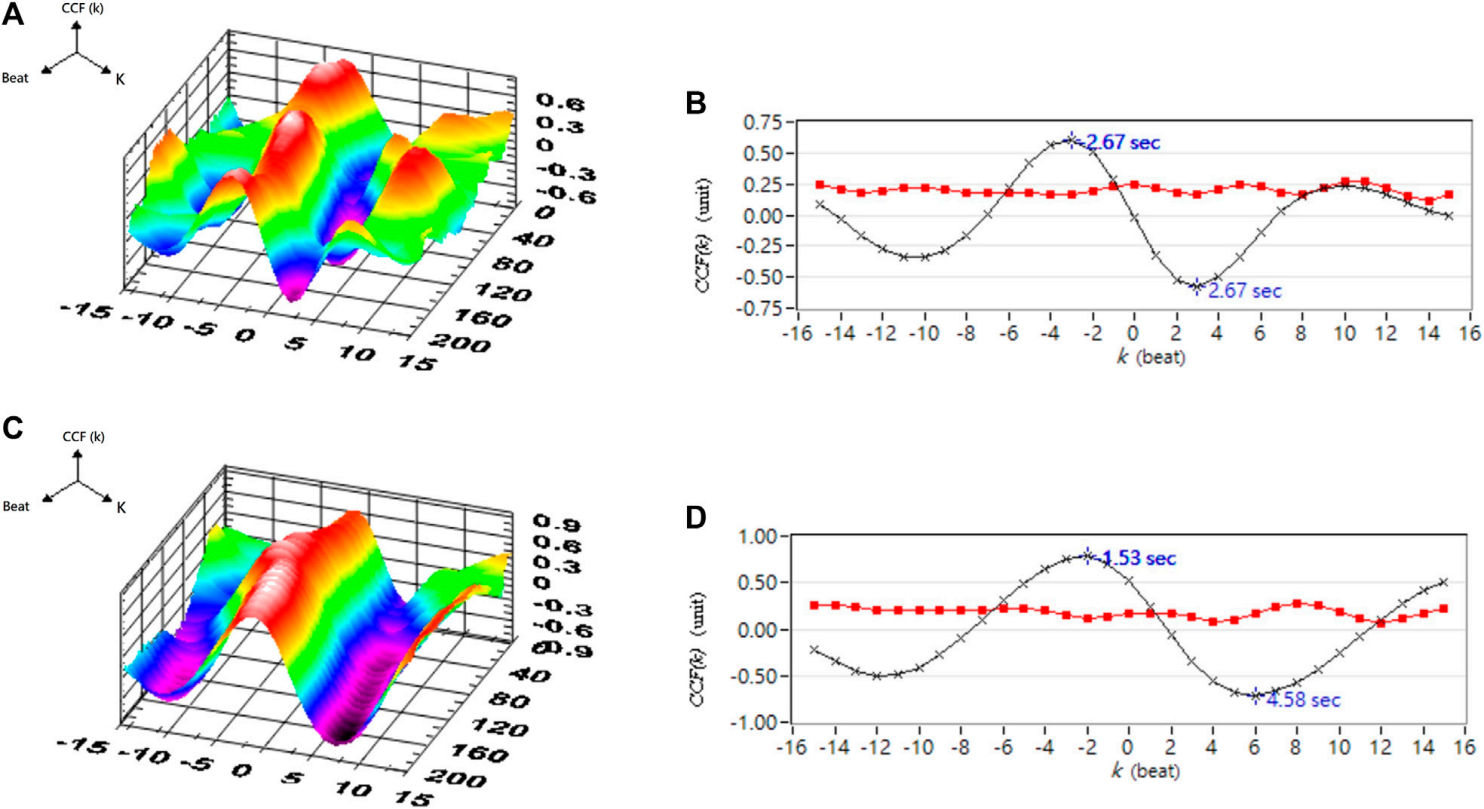
A typical healthy person’s blood pressure and cerebral blood flow correlation analysis. **(A)** 3D representative CCF figures in supine position. **(B)** 2D representative figures of CCF in supine position. **(C)** 3D representative figures of CCF in head-up tilt position. **(D)** 2D representative figures of CCF in head-up tilt position. CCF(k) is the CCF value in the time indices. The mean (–×–) and standard deviation (–■–) of CCF value in each time index k.

**FIGURE 13:**
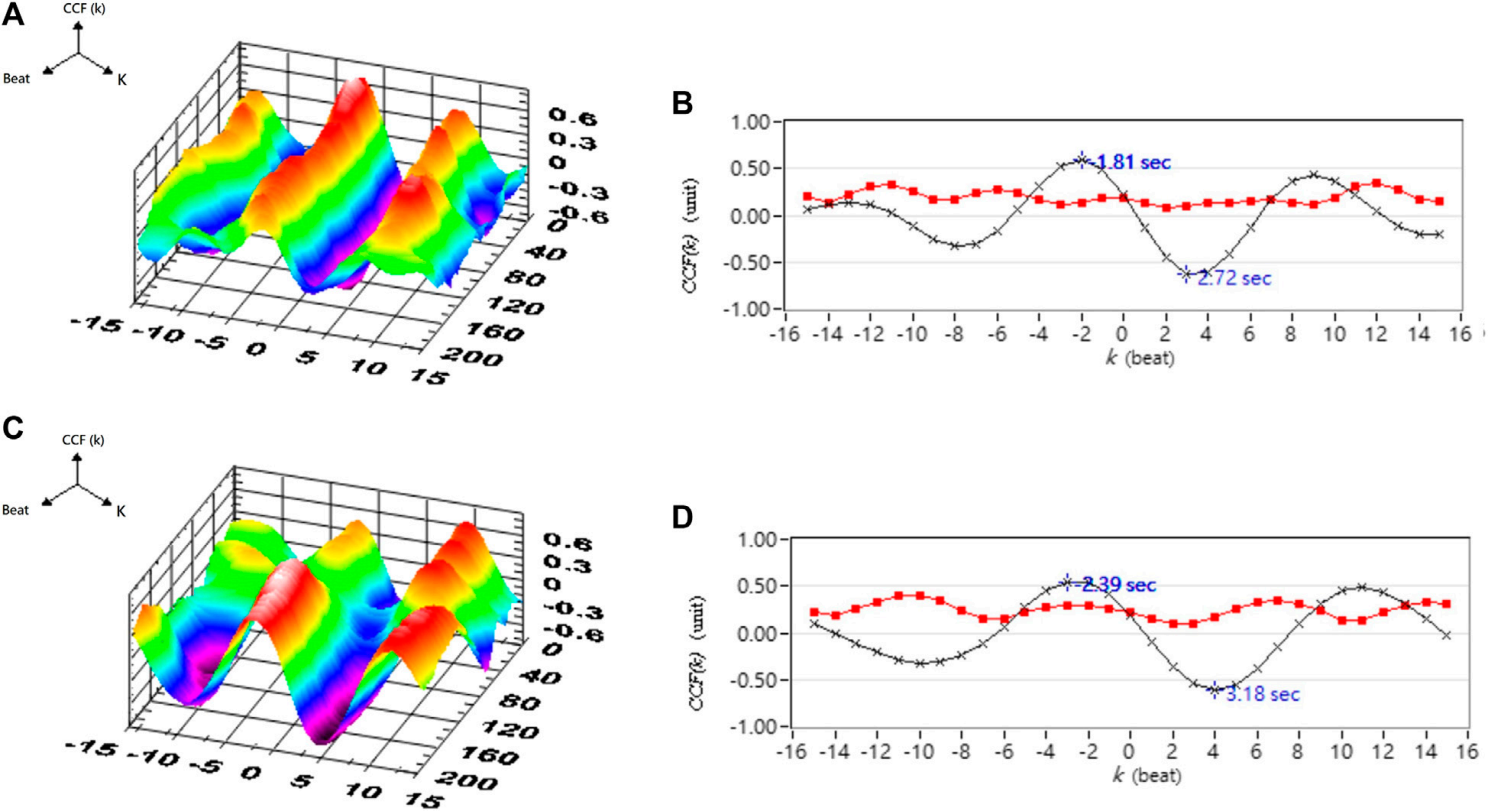
Analysis results of correlation between blood pressure and cerebral blood flow in a typical hypertensive patient. **(A)** 3D representative figures of CCF in supine position. **(B)** 2D representative figures of CCF in supine position. **(C)** 3D representative CCF figures in head-up tilt position. **(D)** 2D representative CCF figures in head-up tilt position. CCF(k) is the CCF value in the time indices. The mean (–×–) and standard deviation (–■–) of CCF value in each time index k.

**FIGURE 14:**
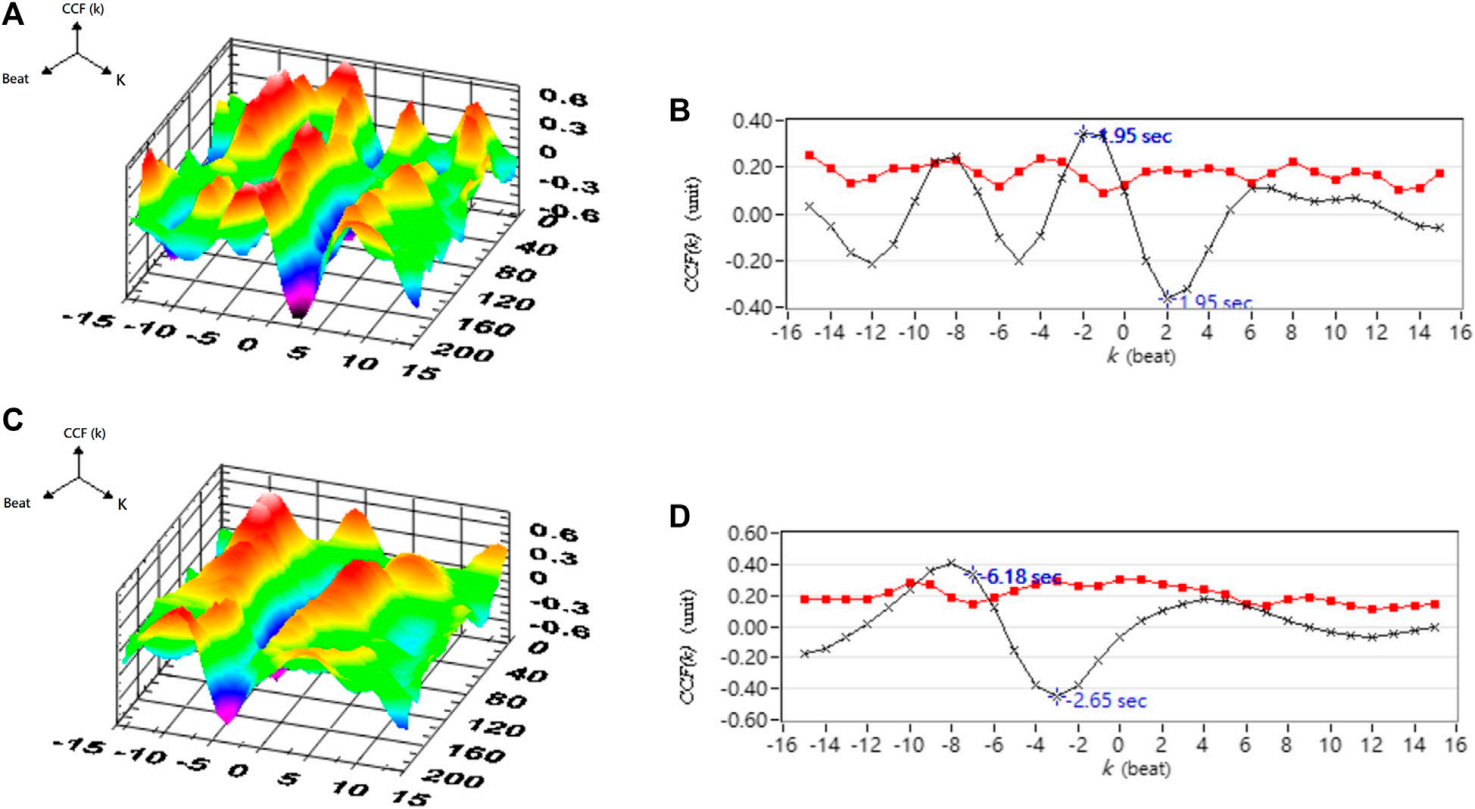
Analysis results for the correlation between blood pressure and cerebral blood flow in a typical stroke patient. **(A)** 3D representative figures of CCF in supine position. **(B)** 2D representative figures of CCF in supine position. **(C)** 3D representative CCF figures in head-up tilt position. **(D)** 2D representative CCF figures in head-up tilt position. CCF(k) is the CCF value in the time indices. The mean (–×–) and standard deviation (–■–) of CCF value in each time index k.

Table 1 shows the correlation analysis results for blood pressure and cerebral blood flow in healthy people, hypertensive patients, and stroke patients. Figure 11 shows the comparison of the maximum CCF differences between blood pressure and cerebral blood flow in healthy people, hypertensive patients, and stroke patients. The results show that as the course of healthy→ hypertension → stroke progresses with the maximum CCF value decreases indicated significantly differences (Healthy vs. Hypertension; Healthy vs. Stroke, *p* < 0.05). That means the relationship between blood pressure and cerebral blood flow decreased, reaching a statistical difference (*p* < 0.05). Figures 12–14 show the correlation analysis between blood pressure and cerebral blood flow in typical healthy people, hypertensive patients, and stroke patients.

**TABLE 1:** Correlation analysis results for blood pressure and cerebral blood flow in each group.

	Supine			Tilting		
	Max CCF	Index	SD	Max CCF	Index	SD
Healthy	0.57	−1.9	015	0.53	−1.6	0.18
Hypertension	0.43*	−2.2	0.16	0.40^	−2.5	0.19
Stroke	0.40**	−0.11	0.20	0.34^^	−3.52	0.22

Max CCF, in supine (Healthy vs. Hypertension, *p < 0.05; Healthy vs. Stroke, **p < 0.05); max CCF, in tilting (Healthy vs. Hypertension, ^*p* < 0.05; Healthy vs. Stroke, ^^*p* < 0.05).

## 4 Discussion

The time domain analysis results indicate that high blood pressure is one of the most important factors influencing the stroke process. Higher blood pressure and blood flow increase the risk for stroke (Fuchs and Ethlton, 2020). Although there is no statistical difference between the average cerebral blood flow and the diastolic cerebral blood flow, it can be clearly observed that the CBFV value of stroke patients is higher than those in healthy and hypertensive patients. Therefore, it can also be inferred that the increase in blood pressure and blood flow is accompanied by a high risk for hypertension and stroke due to hypertrophy and smooth muscle cells remodeling (Yu et al., 2011). On the other hand, the blood vessel resistance index (RI) is a measure of peripheral blood flow resistance. Low vascular resistance has a higher diastolic blood flow velocity characteristic, and will have a lower RI value, and a high vascular resistance has a lower diastolic blood flow velocity characteristic and will induce a higher RI value (Hilz et al., 2004). The results showed that there was no significant difference in RI among the groups in this study. The pulsation index (PI) is a measurement that describes the type of signal waveform. Low intracranial vascular resistance will reduce PI, and rising intracranial pulsations have been found to be related to rising intracranial pressure. The general PI range is between 0.5 and 1.4, less than 0.5 may be an ischemic flow pattern under vascular dilation. PI range greater than 1.5 may indicate a decrease in vascular compliance or an increase in intracranial pressure (Hilz et al., 2004). Because the CVR value in the hypertension group is higher than those in other groups, it would indicate that blood vessel resistance increased and blood vessel characteristics were changed. Moreover, average PI value in hypertension group is 1.37 close to 1.4, it may reveal high blood pressure decrease vascular compliance or increase intracranial pressure (Hilz et al., 2004). From the baroreflex results, it can be inferred that the BRS receptor sensitivity in patients with hypertension and stroke is reduced, and it is unable to effectively sense the changes in blood pressure and regulate the cardiovascular system. If high blood pressure occurs, hypertension exceeds the normal pressure reflex receptor operating range (The average blood pressure is 60–120 mmHg), the individual cannot adjust their heartbeat, blood vessel radius and other factors through the autonomic nervous system to maintain normal cardiovascular system regulation. Therefore, dysfunctional baroreflex and hypertension would lead to stroke risk according to the results and previous studies (Kuusela et al., 2002; Yu et al., 2011; Fuchs et al., 2020).

Chaotic analysis can extract hidden behavior in a system. The K2 value is finite and positive for a chaotic system. Using the BP K2 results in chaotic analysis, it can be speculated that the changes in blood pressure in stroke patients are more unpredictable than in healthy people and hypertensive patients (Grassberger 1983; Zhang 2017). According to previous studies (Eckhardt and Yao, 1993; Rozenbaum et al., 2017), the difference in LE indicated that the blood pressure disturbance in hypertensive patients has a higher change in chaotic behavior and a difference in initial state sensitivity. On the other hand, nonlinear analysis of cerebral blood flow infers that the changes in cerebral blood flow in stroke patients is more unpredictable than in healthy people and hypertensive patients. This revealed that cerebral blood flow disturbance in hypertensive patients has a higher change in chaotic behavior and a difference in initial state sensitivity. Summarizing the chaotic analysis and baroreflex results, due to lower BRS value mean dysfunction baroreflex and it would induced circulation system to be more complicated (Kuusela et al., 2002), it can be inferred that changes in blood pressure and cerebral blood flow in patients with hypertension and stroke lead to higher chaotic behavior and changes in initial state sensitivity. CCF analysis indicated the interaction of circulation subsystems and it showed maximum CCF value decreasing significant in hypertension and stroke group respect to healthy group. This means that blood pressure and cerebral blood flow are gradually not affected by the autoregulation mechanism, and that the buffer between blood pressure and cerebral blood flow is dysfunctional. It can also be speculated that the incidence of stroke is increased.

## 5 Conclusion

This study demonstrated the results from assessing the link change in linear and nonlinear analysis in healthy, hypertensive and stroke groups. The significant differences might indicate high blood pressure would be a critical factor that affects cardiovascular control with regulation function and blood vessel properties in hypertension and stroke subjects. The results from this study revealed the time domain analysis included BP and BFV levels, BRS, CVR and CCF. The nonlinear measures included LE, and K2, which are suitable parameters to explore the hidden components of circulation characteristics and performance in hypertensive and stroke patients. We speculate that an irregular cardiovascular system would tend toward dysfunction in various sub-systems and less predictable behavior. This could be as a measure for detecting and preventing the risk for hypertension and stroke in clinical practice (Faure and Korn, 1998; Rivera-Lara et al., 2017).

## Data Availability

The raw data supporting the conclusion of this article will be made available by the author, without undue reservation.

## References

[B1] Aoi M. C. Hu K. Lo M.-T. Selim M. Olufsen M. S. Novak V. (2012). Impaired Cerebral Autoregulation Is Associated with Brain Atrophy and Worse Functional Status in Chronic Ischemic Stroke. PLoS ONE 7 (10), e46794. 10.1371/journal.pone.0046794 23071639 PMC3469603

[B2] Baumbach G. L. Heistad D. D. (1988). Cerebral Circulation in Chronic Arterial Hypertension. Hypertension 12 (2), 89–95. 10.1161/01.hyp.12.2.89 3044994

[B3] Benjo A. Thompson R. E. Fine D. Hogue C. W. Alejo D. Kaw A. (2007). Pulse Pressure Is an Age-independent Predictor of Stroke Development after Cardiac Surgery. Hypertension 50, 630–635. 10.1161/HYPERTENSIONAHA.107.095513 17785628

[B4] Bolea J. Laguna P. Remartínez J. M. Rovira E. Navarro A. Bailón R. (2014). Methodological Framework for Estimating the Correlation Dimension in HRV Signals. Comput. Math. Methods Med. 2014, 129248. 10.1155/2014/129248 24592284 PMC3926396

[B5] Castro P. Azevedo E. Sorond F. (2018). Cerebral Autoregulation in Stroke. Curr. Atheroscler. Rep. 20, 37. 10.1007/s11883-018-0739-5 29785667

[B6] Chiu C.-C. Yeh S.-J. (2001). Assessment of Cerebral Autoregulation Using Time-Domain Cross-Correlation Analysis. Comput. Biol. Med. 31 (6), 471–480. 10.1016/s0010-4825(01)00015-4 11604152

[B7] Chiu C. C. Yeh S. J. Chen C. H. (2007). Self-organizing Arterial Pressure Pulse Classication Using Neural Networks: Theoretical Considerations and Clinical Applicability. Comput. Biol. Med. 30, 71–88. 10.1016/s0010-4825(99)00023-2 10714443

[B8] Chiu C. C. Yeh S. J. Liau B. Y. (2005). Assessment of Cerebral Autoregulation Dynamics in Diabetics Using Time-Domain Cross-Correlation Analysis. J. Med. Biol. Eng. 25 (2), 53–59.

[B9] Crippa I. A. Subirà C. Vincent J.-L. Fernandez R. F. Hernandez S. C. Cavicchi F. Z. (2018). Impaired Cerebral Autoregulation Is Associated with Brain Dysfunction in Patients with Sepsis. Crit. Care 22, 327. 10.1186/s13054-018-2258-8 30514349 PMC6280405

[B10] Eckhardt B. Yao D. (1993). Local Lyapunov Exponents in Chaotic Systems. Physica D: Nonlinear Phenomena 65, 100–108. 10.1016/0167-2789(93)90007-n

[B11] Fan X. Li X. Yin J. (2019). Dynamic Relationship between Carbon price and Coal price: Perspective Based on Detrended Cross-Correlation Analysis. Energ. Proced. 158, 3470–3475. 10.1016/j.egypro.2019.01.925

[B12] Faure P. Korn H. (1998). A New Method to Estimate the Kolmogorov Entropy from Recurrence Plots: its Application to Neuronal Signals. Physica D: Nonlinear Phenomena 122, 265–279. 10.1016/s0167-2789(98)00177-8

[B13] Fuchs F. D. Whelton P. K. (2020). High Blood Pressure and Cardiovascular Disease. Hypertension 75 (2), 285–292. 10.1161/hypertensionaha.119.14240 31865786 PMC10243231

[B14] Gao J. Hu J. Tung W.-w. (2011). Facilitating Joint Chaos and Fractal Analysis of Biosignals through Nonlinear Adaptive Filtering. PLoS ONE 6 (9), e24331. 10.1371/journal.pone.0024331 21915312 PMC3167840

[B15] Grassberger P. Procaccia I. (1983). Estimation of the Kolmogorov Entropy from a Chaotic Signal. Phys. Rev. A. 28 (4), 2591–2593. 10.1103/physreva.28.2591

[B16] Grassberger P. Procaccia I. (1983). Measuring the Strangeness of Strange Attractors. Physica D: Nonlinear Phenomena 9, 189–208. 10.1016/0167-2789(83)90298-1

[B17] Hilz M. J. Kolodny E. H. Brys M. Stemper B. Haendl T. Marthol H. (2004). Reduced Cerebral Blood Flow Velocity and Impaired Cerebral Autoregulation in Patients with Fabry Disease. J. Neurol. 251 (5), 564–570. 10.1007/s00415-004-0364-9 15164189

[B18] Karemaker J. M. Wesseling K. H. (2008). Variability in Cardiovascular Control: The Baroreflex Reconsidered. Cardiovasc. Eng. 8, 23–29. 10.1007/s10558-007-9046-4 18041583

[B19] Kuusela T. A. Jartti T. T. Tahvanainen K. U. O. Kaila T. J. (2002). Terbutaline-Induced Heart Rate and Blood Pressure Changes. Am. J. Physiol. Heart Circ. Physiol. 282, H773–H781. 11788429 10.1152/ajpheart.00559.2001

[B20] Laurent S. Katsahian S. Fassot C. Tropeano A.-I. Gautier I. Laloux B. (2003). Aortic Stiffness Is an Independent Predictor of Fatal Stroke in Essential Hypertension. Stroke 34, 1203–1206. 10.1161/01.STR.0000065428.03209.64 12677025

[B21] Laurent S. p. Boutouyrie P. (2005). Arterial Stiffness and Stroke in Hypertension. CNS Drugs 19 (1), 1–11. 10.2165/00023210-200519010-00001 15651901

[B22] Liau B.-Y. Yeh S.-J. Chiu C.-C. Tsai Y.-C. (2008). Dynamic Cerebral Autoregulation Assessment Using Chaotic Analysis in Diabetic Autonomic Neuropathy. Med. Bio Eng. Comput. 46, 1–9. 10.1007/s11517-007-0243-5 17874153

[B23] Liau B. Y. Chiu C. C. Yeh S. J. (2010). Assessment of Dynamic Cerebral Autoregulation Using Spectral and Cross-Correlation Analyses of Different Antihypertensive Drug Treatments. J. Med. Biol. Eng. 30 (3), 169–176.

[B24] Ma H. Guo Z.-N. Liu J. Xing Y. Zhao R. Yang Y. (2016). Temporal Course of Dynamic Cerebral Autoregulation in Patients with Intracerebral Hemorrhage. Stroke 47, 674–681. 10.1161/STROKEAHA.115.011453 26846864

[B25] Oeinck M. Neunhoeffer F. Buttler K.-J. Meckel S. Schmidt B. Czosnyka M. (2013). Dynamic Cerebral Autoregulation in Acute Intracerebral Hemorrhage. Stroke 44, 2722–2728. 10.1161/STROKEAHA.113.001913 23943213

[B26] Pringle E. Phillips C. Thijs L. Davidson C. Staessen J. A. de Leeuw P. W. (2003). Systolic Blood Pressure Variability as a Risk Factor for Stroke and Cardiovascular Mortality in the Elderly Hypertensive Population. J. Hypertens. 21, 2251–2257. 10.1097/00004872-200312000-00012 14654744

[B27] Rivera-Lara L. Zorrilla-Vaca A. Geocadin R. G. Healy R. J. Ziai W. Mirski M. A. (2017). Cerebral Autoregulation-Oriented Therapy at the Bedside. Anesthesiology 126 (6), 1187–1199. 10.1097/aln.0000000000001625 28383324

[B28] Rivera-Lara L. Zorrilla-Vaca A. Geocadin R. Ziai W. Healy R. Thompson R. (2017). Predictors of Outcome with Cerebral Autoregulation Monitoring. Crit. Care Med. 45 (4), 695–704. 10.1097/CCM.0000000000002251 28291094

[B29] Roth G. A. Mensah G. A. Johnson C. O. Addolorato G. Ammirati E. Baddour L. M. (2020). Global Burden of Cardiovascular Diseases and Risk Factors, 1990-2019: Update from the GBD 2019 Study. J. Am. Coll. Cardiol. 76 (5), 2982–3021. 10.1016/j.jacc.2020.11.010 33309175 PMC7755038

[B30] Rozenbaum E. B. Ganeshan S. Galitski V. (2017). Lyapunov Exponent and Out-Of-Time-Ordered Correlator's Growth Rate in a Chaotic System. Phys. Rev. Lett. 118, 086801. 10.1103/PhysRevLett.118.086801 28282154

[B31] Shekhar S. Liu R. Travis O. K. Roman R. J. Fan F. (2017). Cerebral Autoregulation in Hypertension and Ischemic Stroke: a Mini Review. J. Pharm. Sci. Exp. Pharmacol. 2017 (1), 21–27. 29333537 PMC5765762

[B32] Tiecks F. P. Lam A. M. Aaslid R. Newell D. W. (1995). Comparison of Static and Dynamic Cerebral Autoregulation Measurements. Stroke 26 (6), 1014–1019. 10.1161/01.str.26.6.1014 7762016

[B33] WHO CVD risk chart working group (2019). World Health Organization Cardiovascular Disease Risk Charts: Revised Models to Estimate Risk in 21 Global Regions. Lancet Glob. Health 7 (10), e1332–e1345. 10.1016/S2214-109X(19)30318-3 31488387 PMC7025029

[B34] WHO (2003), International Society of Hypertension Writing Group. J. Hypertens. 21 (11), 1983–1992. 14597836 10.1097/00004872-200311000-00002

[B35] Xiong L. Liu X. Shang T. Smielewski P. Donnelly J. Guo Z.-n. (2017). Impaired Cerebral Autoregulation: Measurement and Application to Stroke. J. Neurol. Neurosurg. Psychiatry 88, 520–531. 10.1136/jnnp-2016-314385 28536207

[B36] Yu J.-G. Zhou R.-R. Cai G.-J. (2011). From Hypertension to Stroke: Mechanisms and Potential Prevention Strategies. CNS Neurosci. Ther. 17, 577–584. 10.1111/j.1755-5949.2011.00264.x 21951373 PMC6493871

[B37] Zhang X. D. (2017). “Entropy for the Complexity of Physiological Signal Dynamics,”. Editor Shen B. (Springer Nature Singapore Pte Ltd.), Adv. Exp. Med. Biol., 1028, 39–53. 10.1007/978-981-10-6041-0_3 29058215

